# Electrical Disturbances in Terms of Methods to Reduce False Activation of Aerial Fire Protection Systems

**DOI:** 10.3390/s22208059

**Published:** 2022-10-21

**Authors:** Andrzej Żyluk, Mariusz Zieja, Andrzej Szelmanowski, Justyna Tomaszewska, Magdalena Perlińska, Krzysztof Głyda

**Affiliations:** 1 Air Force Institute of Technology, 01-494 Warsaw, Poland; 2 Faculty of Aviation, Polish Air Force University, 08-521 Deblin, Poland; 3 Royal-Star Aero, 39-300 Mielec, Poland

**Keywords:** fire protection system, aircraft AN-28, SSP-FK-BI executive blocks

## Abstract

The paper presents an analysis of false triggers of fire protection systems installed on aircraft. They not only cause task interruption but also have a direct impact on flight safety, forcing the crew to land in a risky area. Simulation models of electronic actuators were developed to determine the conditions under which false alarms occur. Testing of the simulation models was carried out in the computational package Matlab-Simulink and Circum-Maker for different electrical disturbance generation conditions. The simulation of overvoltage, voltage drops and voltage decays in the on-board electrical network supplying the fire protection system, occurring during the start-up of aircraft engines and during the switching on and off of on-board high-power devices, was studied. The conducted studies have practical applications since the simulation results are an important element for planning experimental tests of the SSP-FK-BI executive blocks under electrical disturbance conditions. Based on the simulation and experimental studies, the conditions causing false tripping of the fire protection system and the parameters for selected disturbance factors have been determined.

## 1. Introduction

Fire on board an aircraft is a very serious hazard; all precautions must be taken to minimize the risk of a fire starting. If a fire does occur, there must be adequate fire protection on the aircraft. Fire protection includes the means of both detecting and extinguishing the fire. The subject of fire protection theory is a branch of engineering in its own right [1].

Fire detection and protection exist in the engines, auxiliary power unit (if installed) and cargo compartments of modern transport aircraft. Smoke detectors are installed in lavatories, with automatic fire extinguishers in the waste bins. Other parts of the aircraft are unprotected. In the unprotected areas, fire detection depends upon flight crew and the cabin crew involvement.

Prevention of a fire, while desirable, is not possible in all cases. Consequently, consideration of detecting and fighting fire must be included in the mitigations.

As opposed to other aircraft installations, such as hydraulic or fuel systems, the fire protection system does not work during normal aircraft flight. However, it must be ready to respond to emergency situations. The reliability of the operation of this system therefore requires the use of appropriate design solutions to fulfill the tasks specified for them in situations that threaten the safety of flight operations.

Sometimes, unfortunately, the operation of fire protection systems does not proceed as expected. Catastrophic events can occur as a result of malfunctions, failure to signal a fire or false alarms [2,3].

These days, the fire detection systems used, for example in the cargo hold of an aircraft, are based on smoke detection. The smoke detectors in modern aircraft are mainly photoelectric particle detectors that reliably detect smoke, but also dust, fog and most other small particles. Fake alarms caused by these contaminants can be very costly for airlines, as they can cause unnecessary flight diversions. To minimize this expense, a new approach to detecting fires on board aircraft is needed.

The authors of article [4] describe an innovative fire detection system developed by Goodrich’s Advanced Sensors Technical Centre. The system uses multiple sensors with different technology to allow distinction between real fire events and false alarm triggers. The system uses infrared imaging and multiple distributed chemical sensors and smoke detectors that transmit data to a digital signal processor. The processor integrates data from the chemical sensors, smoke detectors and processed imagery to determine whether a fire (or potential fire) is present. Decision-making algorithms analyze all these data in real time and make a final decision about the presence of a fire [5]. Other authors show that multiple sensing technologies are crucial for reducing false alarms in fire protection systems on the aircraft board [6].

The method used in this publication was developed at the Air Force Institute of Technology and is based on the knowledge of fire protection systems. The method makes it possible to determine the parameters of fire protection system components, such as sensors and BI-2A blocks operated on AN-28 and Mi-8, which, if exceeded, can cause false tripping. This method will be presented in detail later in the paper.

The main contributions of this paper can be summarized as follows:

- Simulation of DPS and DTBG sensor operation under fluctuating thermal states;
- Since the false triggering of fire protection systems is almost impossible to test in an aircraft, simulation and experimental studies are practically the only possible methods to study the parameters that can affect the malfunctioning of the systems;
- Construction of a test platform and testing the behavior of SSP-FK-BI executive blocks under electrical disturbance conditions.

## 2. Literature Review

### 2.1. The Fire Protection System

The fire protection system on board can be in the form of a simple fire-extinguishing system (activated manually by the pilot) [7,8] or a complex automatic fire detection and suppression system [9,10,11].

Essentially, a fire protection system is designed to automatically detect and signal the occurrence of a fire in a supervised compartment of the aircraft (engine area, pressure regulator, heating furnace, passenger cabin, cockpit) and to extinguish the fire outbreak in automatic mode (the so-called first order of extinguishing) or manually by the pilot or crew members (the so-called second order of extinguishing).

The main tasks for the fire protection system are [12,13]:

- Detecting fire outbreaks in terms of the occurrence of a flame (air ionisation), increased ambient air temperature or a sufficiently rapid change in air temperature in the vicinity of the fire detector (thermoelectric force);
- Indication of the occurrence of fire, indicating the supervised compartment in which the system has detected an outbreak of fire and the technical condition and the number of cylinders of extinguishing fluid remaining in service;
- Preparing the fire-fighting system for use by selecting the appropriate operating ranges on the fire-fighting system control panel.

In both western and eastern solutions, the fire protection system contains two functional subsystems: the system for detecting and signaling the occurrence of a fire (fire detectors and control blocks) and the fire extinguishing system (buses, valves, extinguishing cylinders). Fire detection can be realized using various principles: the thermoelectric phenomenon, consisting of the generation of thermoelectric force under conditions of temperature change in the supervised compartment (e.g., DPS and DTBG thermoelectric sensors), or the emergence of electric conduction conditions on the powered part of the signaling system (e.g., IS-5 ionization sensors).

On commercial light aircraft (avionettes), fire protection equipment consists of an extinguishing cylinder, manually activated by the pilot in the event of a fire outbreak [14,15]. This type of solution is used, among others, on a multi-role aircraft designed primarily for sports aviation and single-seat aircraft designed for agriculture.

On large commercial (passenger or transport) aircraft, a fire suppression system including a fire detection system and a fire extinguishing system is used. For example, on a Boeing 737 aircraft, the fire suppression system includes: overheat sensors (in case of temperature rise), fire sensors (in case of a fire outbreak), control blocks, a system monitor and a control panel for the crew [16]

Analyzing the available literature, both general as well as specialized, it can be concluded that there is a lack of detailed information characterizing the properties of aerial firefighting systems. Due to the information being proprietary to a particular company or institution, there is a lack of parameter values in these studies in terms of high-tech systems.

### 2.2. False Alarms Definition

False alarms are—in fire safety engineering terms—fire alarms in the absence of an actual fire condition. They are associated with information exchange and occur as a side effect of system technologies and the alarm process. In many cases, fires are associated with significant damage. Since time is a critical factor, detection is often carried out by installed fire detection and alarm systems (FDAS). Having highly sensitive sensors in fire detectors allows fires to be identified at an early stage, but this also makes FDAS susceptible to false alarms [17].

False warnings are often generated from sensors in the cargo area of aircraft, less frequently from the passenger cabin. Two types of detectors are used there: photoelectric, which works based on the principle of light beam scattering, and ionization using the law of voltage/current drop [18].

### 2.3. Consequences of False Alarms

The serious consequences that may occur in case of false fire alarms may be proved by the incident of the Boeing 777-39LER aircraft of the Chinese Air China, flying from Beijing to Los Angeles. Due to a fire alarm in the aircraft’s hold, the crew was forced to make an emergency landing at Anadyr Airport in Russia and evacuate 188 passengers. After landing and evacuation, the inspection of the cargo hold did not detect any signs of fire. The probable cause, confirmed by the carrier, was a false fire signal generated from sensors located in the cargo hold of the aircraft [19].

In the engine nacelles, the sensors placed can also send false signals about the occurrence of fire under the influence of various external factors. Depending on the fire suppression system, this may even lead to automatic activation of the fire suppression system, which is beyond the control of the aircraft crew. As an example, an Air France incident occurred on 2 February 2018 when, during a flight on a B777-300 aircraft from Hong Kong to Paris, the crew received information that a fire had been detected in the engine. The consequence was a return to Hong Kong airport, which involved the evacuation of passengers and the arrival of emergency services. However, the assistance was unnecessary, because after landing it turned out that the signal about the existence of fire in the engine turned out to be false. The probable cause of the failure of the fire detection system was a faulty sensor [20].

The next example of a false tripping of the fire detection system on an aircraft may be the LOT Polish Airlines incident of 16 January 2019. In a Bombardier Q400 aircraft, during the approach to landing at EPPO airport, the crew received a signal that a fire had been detected in one of the engines, after which they proceeded to perform emergency procedures, including shutting down the engine in question and activating fire extinguishers. After an unsafe landing on one running engine, an inspection of the shutdown engine was made. No signs of fire were detected at that time. During an inspection of the aircraft’s fire suppression system, a fault was located in connector 2600-J24, the probable cause of which was a short circuit caused by moisture and was thus the source of the false fire signal [21,22].

During flight, the impact of a false fire warning is significant and can result in actions including flight diversion, declaration of an emergency requiring immediate landing, evacuation of passengers and replacement or repair of faulty system components. All of these actions not only lead to disruption of flight operations but also result in increased aircraft operating costs. As long as the crew is unable to distinguish between true and false fire warnings, they must follow the prescribed procedures.

## 3. Analysis of the Malfunction of the Fire Protection System of the AN-28 Aircraft

The fire protection system of the An-28 aircraft is designed to detect and suppress fires in the engine nacelles and crew cabin. Unfortunately, like any system, it shows signs of malfunctioning of individual system components depending on operating conditions. Certain factors such as changes in ambient temperature, humidity or voltage drops in the onboard network can affect the negative functioning of selected components of this system.

In the case of the AN-28 aircraft fire protection system, whose operation is based on the first and second order of fire extinguishing, the proper functioning of the system components responsible for detecting and signaling a fire is particularly important, as the first order is activated automatically. Therefore, in the event of a false fire warning, the crew is not able to decide on manual control of the extinguishing process.

Incorrect fire signaling or lack of signaling during flight operations are significant problems that can lead to serious incidents.

In this paper, the analysis of damage to the fire-suppression system of the AN-28 aircraft was based on data obtained from the process of operation of eight aircraft used in Poland in 2015–2019. The damage found to elements of the AN-28 aircraft fire protection system is presented in Table 1.

The operating data in Table 1 show that the most frequent failures are the DPS transmitters, located in the engine nacelles, and the BI-2A executive block. Failures of these components are regarded as important defects, as correct signaling of the occurrence of a fire depends on their failure.

Due to the limited number of examples of failures of the An-28 aircraft’s fire protection system components included in the paper, in the following section, only the operation of the DPS transmitters and the BI-2A executive block are analyzed in terms of the causes of their false activation.

## 4. Fire Protection Installation of the AN-28 Aircraft

The fire protection system on the An-28 aircraft is designed to signal and extinguish fires in the engine nacelles and cabin.

The fire protection system consists of a stationary fire protection system and a portable hand-held fire extinguisher located in the cabin. The stationary fire protection system includes a signaling and control system and a fire extinguishing system. One of the system’s extinguishers is located in each engine nacelle. The fire extinguishing control and the control of the correct operation of the system is carried out from the cockpit [23].

The operation of the fire protection system assumes two orders of fire extinguishing in the engine nacelles—the first is carried out automatically and, if the fire is not extinguished, the units of the second order of extinguishing are manually switched on.

The alarm and control system makes it possible to signal the onset and disappearance of a fire, to control the readiness of the fire extinguishing system, to signal the activation of fire extinguishers and to allow automatic and manual fire extinguishing. The system consists of:

- Fire alarm system SSP-2A series 2,
- Elements for controlling and monitoring the correct operation of the system,
- Electrical wiring and fittings,
- Fire extinguisher pyro-heads.

The signaling system is controlled by a BI-2A series 2 block, which is in the roof of the passenger cabin, and the system’s actuators are pyronabuses, two of which are located in each of the fire extinguisher pyro-heads. Detectors signaling the occurrence of a fire are built into the engine nacelles in the areas most exposed to fire. The system is supplied with 27 V DC. A PM fuse is built into the power supply system, which is located on the right-hand panel, and the switchgear is located in the relay box [24].

### 4.1. The SSP-2A Series 2 Fire Alarm System

The SSP-2A signaling system comprises:

- 18 DPS transmitters,
- 18 SSP-2I-RM sockets,
- BI-2A executive block series 2.

The principle of operation of the SSP-2A series 2 signaling system makes use of the phenomenon of post-electromotive force in the sensors during a temperature change under standard operating conditions. The resistance of the wires connecting a single group of three detectors to the block must not be higher than 10 Ω. These wires from the block to one group of detectors and from the detectors to the block are laid in pairs with a turn pitch of 100 mm, which, together with the shielding of the wires, prevents accidental tripping of the system [24].

### 4.2. Description and Principle of Operation of the DPS Transmitters

The fire protection system of the An-28 aircraft includes 18 DPS transmitters, nine per engine nacelle, mounted in SSP-2I-RM sockets. The purpose of the transmitters is to detect and signal the occurrence of post-fire in the engines [21].

**Figure 1:**
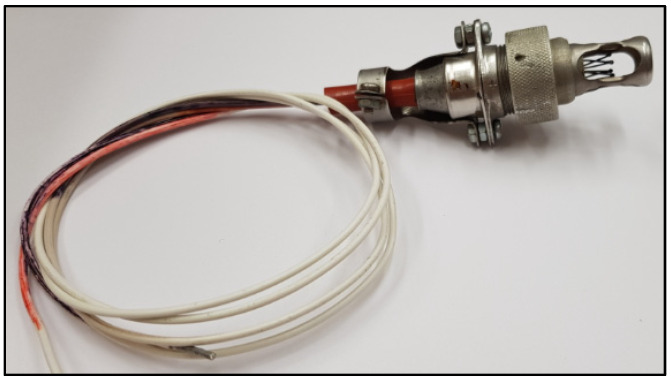
View of the DPS transmitter.

The DPS transmitters are thermoelectric fire detectors, which consist of a single open junction system, protected by a mechanical shield, and covered junctions, located inside the sensor [25]. Figure 1 shows the view of a DPS sensor.

**Figure 2:**
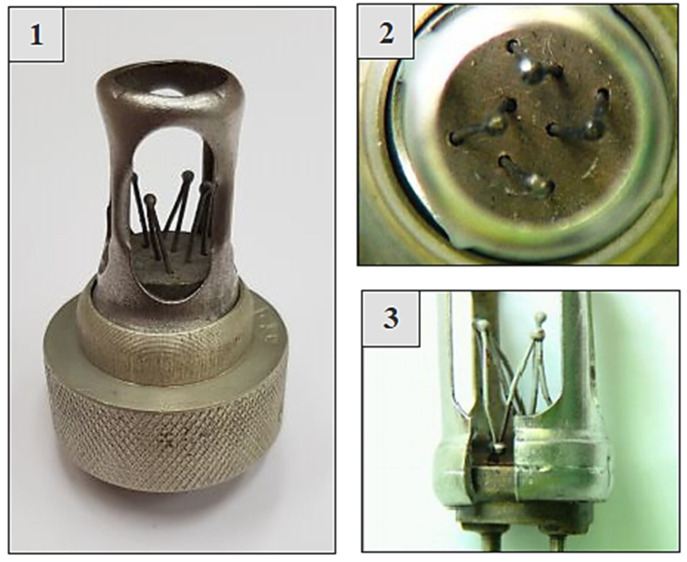
View of uncovered joints—1,2 and covered joints—3 of the DPS sensor.

When the environmental temperature at the emitter location increases at a rate of min. 2–4 °C per second, while causing the sensor to heat up to temperatures between 180 and 400 °C, a thermoelectric force is generated in the transmitter proportional to the temperature difference between the temperature of the exposed welds and the temperature of the covered welds. Figure 2 shows a view of the exposed and covered welds of the DPS transmitter.

A voltage signal, which is the result of thermoelectric forces generated in exposed and covered welds, is supplied from the DPS transmitter to the BI-2A actuating block and compared with the permissible reference voltage value. If this value is exceeded, a signal appears at the output of the BI-2A block, which activates the fire signaling and extinguishing systems [8].

### 4.3. The Simulation Model of Thermoelectric Fire Sensors

A full description of the mathematical model of the operation of a DPS-type thermoelectric fire sensor requires an analysis of the instantaneous temperature distributions of the individual exposed joints and covered joints (as well as electrical conductors and insulating materials connecting the joints placed inside the joint) using thermal conductivity equations, taking into account the delays in the heating and cooling of the joints.

The method adopted in this work is to use the approach used in automation [26] and describe the operation of the DPS thermoelectric sensor as a first-order inertial element with a known value of the time constant conditioning the rate of increase of the generated thermoelectric force under the conditions of heating of the sensor’s exposed joints.

A simplified mathematical model describing the operation of a thermoelectric DPS fire sensor, with one crown of exposed joints, can be presented using the dependency [27]:

$$U_{DPS}^{}(s)=\left\{K_{DPS}^{}\left[\frac{1}{\tau_{SO}\cdot s+1}\cdot T_{ENV}(s)-\frac{1}{\tau_{SZ}\cdot s+1}\cdot T_{CON}(s)\right]\right\}$$

where:

- $U_{DPS}\left(s\right)$—Laplace operator transform of the thermoelectric force generated in a DPS sensor with a single weld crown;
- $K_{DPS}$—scaling factor of the DPS sensor;
- $T_{ENV}\left(s\right)$—Laplace operator transform of the temperature of the air surrounding the DPS sensor, acting directly on the exposed welds by heating or cooling them;
- $T_{CON}\left(s\right)$—Laplace operator transform of the electrical junction temperature of the DPS sensor, acting indirectly on the covered welds and delaying their heating or cooling;
- $\tau_{SO}$—time constant of uncovered joints;
- $\tau_{SZ}$—time constant of covered joints (built into the joint).

According to that model, it is possible, among other possibilities, to study the influence of delays in the heating and cooling of individual joints of the DPS sensor on the characteristics of the signal and the maximum value of the thermoelectric power generated therein for selected cases of change in the temperature of the environment surrounding the exposed joints. The results of such tests make it possible to assess the possibility of sub-threshold signal generation from the fire sensor causing false tripping and activation of the SSP-FK fire protection system at reduced supply voltage.

Similarly, as with the DPS sensor, a complete description of the mathematical model for the operation of the DTBG-type thermoelectric fire sensor requires an analysis of the instantaneous temperature distributions of individual joints using the thermal conductivity equations.

A simplified mathematical model describing the operation of a DTBG thermoelectric fire sensor, with two crowns of exposed joints, can be presented using the relationship [27]:

$$U_{DTBG}^{}(s)=\left\{\begin{array}{l} K_{1}^{}\left[\frac{1}{\tau_{SO1}\cdot s+1}\cdot T_{ENV}(s)-\frac{1}{\tau_{SZ1}\cdot s+1}\cdot T_{CON}(s)\right]+\\ +K_{2}^{}\left[\frac{1}{\tau_{SO2}\cdot s+1}\cdot T_{ENV}(s)-\frac{1}{\tau_{SO1}\cdot s+1}\cdot T_{ENV}(s)\right] \end{array}\right\}$$

where:

- $U_{DTBG}\left(s\right)$—Laplace operator transform of the thermoelectric force generated in a DTBG sensor with a double crown of bare joints;
- $K_{1}$—scaling factor of DTBG sensor for the first crown of bare joints;
- $K_{2}$—DTBG sensor scaling factor for the second crown of exposed joints;
- $T_{ENV}\left(s\right)$—Laplace operator transform of the temperature of the environment surrounding the DTBG sensor, acting directly on the I crown and II crown exposed welds by heating or cooling them;
- $T_{CON}\left(s\right)$—Laplace operator transform of the electrical junction temperature of the DTGB sensor, acting indirectly on the covered welds and delaying their heating or cooling;
- $\tau_{SO1}$—time constant of uncovered joints I crown;
- $\tau_{SO2}$—time constant of uncovered joints II crown;
- $\tau_{SZ}$—time constant of covered joints (built into the joint).

**Figure 3:**
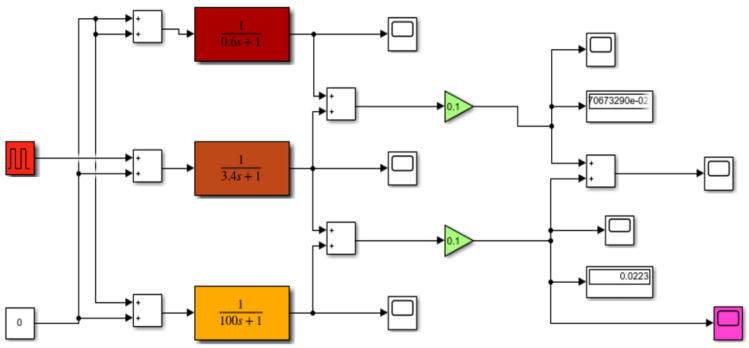
A schematic of the simulation model of the DPS sensors.

The example simulation model (Figure 3), developed in the Matlab-Simulink package, includes the operation of a DPS sensor with a single crown of exposed joints and the operation of a DTBG sensor with a double crown of exposed joints.

To set the static properties of the sensor, the elements describing the amplifications (values of 0.1 mV/C) are used, while to set the dynamic properties, the elements describing the transmittances of the welds with time constants (3.4 s for the I crown and 0.6 s for the II crown) are used. In addition, there is a member describing the transmittance of covered welds placed in the sensor joint (with a value of 100 s).

The model allows the input of preset ambient temperature waveforms (characterizing the temperature conditions of the selected season), heating temperature (temperature rise after switching on the KO-50 heating furnace or EWU exhaust gas scatterers) and cooling temperature (temperature drop with increasing flight altitude during aircraft flight execution).

### 4.4. Description and Principle of Operation of BI—2A Executive Block

**Figure 4:**
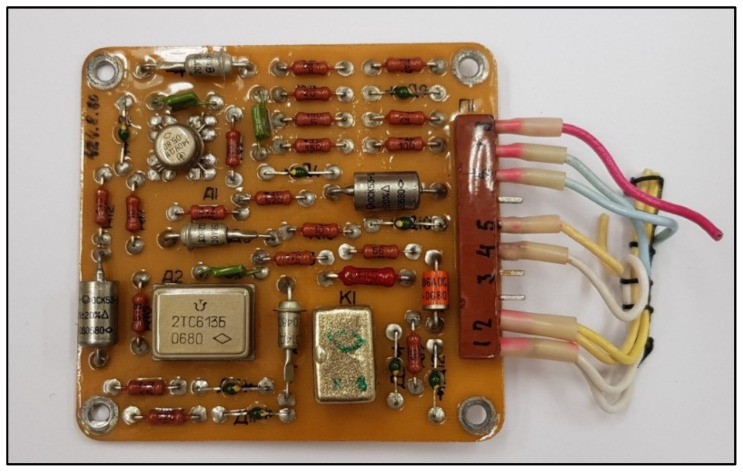
Amplifier board of the BI-2A block seen from the side of the electronic components.

**Figure 5:**
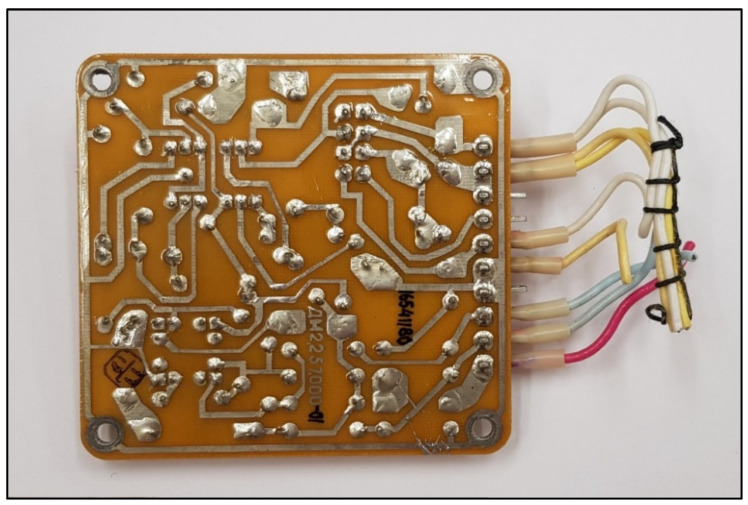
PCB of the BI-2A block of amplifiers from the side of the connections of the electronic components.

The BI-2A executive block is the control element for the fire alarm system, located in the passenger cabin of the aircraft, and consists of six electronic boards, i.e., amplifier boards, made on printed circuits with single-sided mounting, which are fixed on a base. To avoid the possibility of the system tripping itself, the actuator block must be reliably earthed [17]. Figure 4 and Figure 5 show a single amplifier board of the BI-2A block.

### 4.5. A Simulation Model of an Amplifier Board

The aim of the simulation studies of the selected electrical circuit models of the SSP-FK-BI execution blocks (developed in the Circuit-Maker computing package) was to verify the hypothesis of the possibility of their false tripping due to the influence of interference pulses occurring in the power and control circuits of the amplifier boards or the occurrence of short circuits between their internal electrical circuits [28].

To model the system response conditions from disturbances in the electrical power supply, a case was adopted [28] for investigating the possibility of false tripping of the system under conditions of a rapid drop in supply voltage. The study of the influence of temporary supply voltage drops, due to the characteristics of the US1 comparator input signal comparison channel, required additional modeling of the operation of the DPS fire sensors and DTBG, including the determination of the course of the fire signal under conditions of sudden heating of the sensor. The next two cases include the occurrence of momentary overvoltage (for a dry block, for which the insulation resistance between electrical circuits on the amplifier board meets the expectations).

**Figure 6:**
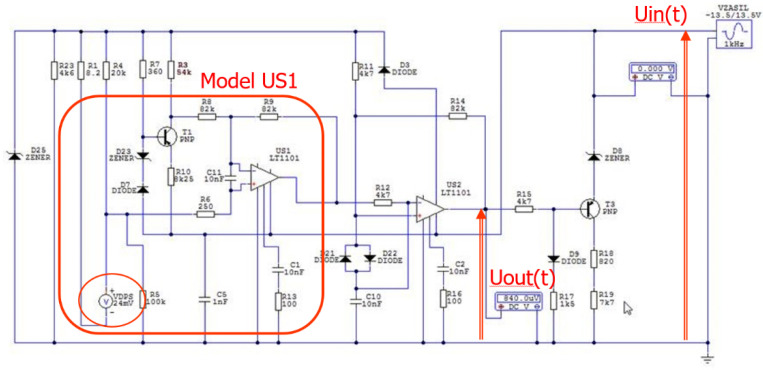
Conceptual diagram of the US1 amplifier model in the SSP−FK−BI execution block.

The example case in the modeling of the operation of the SSP-FK system, in which false tripping occurs, involves the occurrence of momentary drops in the supply voltage during the occurrence of a sub-threshold signal from the DPS sensor (Figure 6).

The operation of the input path of the amplifier board of the SSP−FK−BI block in such a condition consists of the fact that the signal at the inverting input of the US1 amplifier operating in the comparator circuit (constituting the reference voltage) is exceeded by the input voltage from the DPS sensor circuit, fed to the non-inverting input of the comparator.

The voltage at the output of the US1 comparator under the conditions of the occurrence of momentary voltage drops of the supply voltage to the amplifier board and the simultaneous occurrence of a sub-threshold signal from the DPS fire sensor can be presented in the following form [29]:

$$U_{US1}(s)=K_{ZAST}\left[K_{DPS}\cdot U_{DPS}(s)-G_{ZAST}(s)\cdot U_{IN}(s)\right]$$

where:

- $U_{US1}\left(s\right)$—Laplace transform of the voltage at the control output of comparator US1 causing false tripping of the system during the subthreshold signal from the DPS fire sensor;
- $K_{ZAST}$—substitute gain of comparator US1;
- $K_{DPS}$—substitute amplification of the signal from the DPS fire sensor;
- $U_{DPS}$—Laplace transform of the voltage generated at the DPS fire sensor;
- $G_{ZAST}$—substitute transmittance of the signal processing path from the amplifier board supply point to the input of comparator US1;
- $U_{IN}$—Laplace transform of the supply voltage of the amplifier board with instantaneous voltage drops.

## 5. Methods of Experiment

### 5.1. Experimental Setup

**Figure 7:**
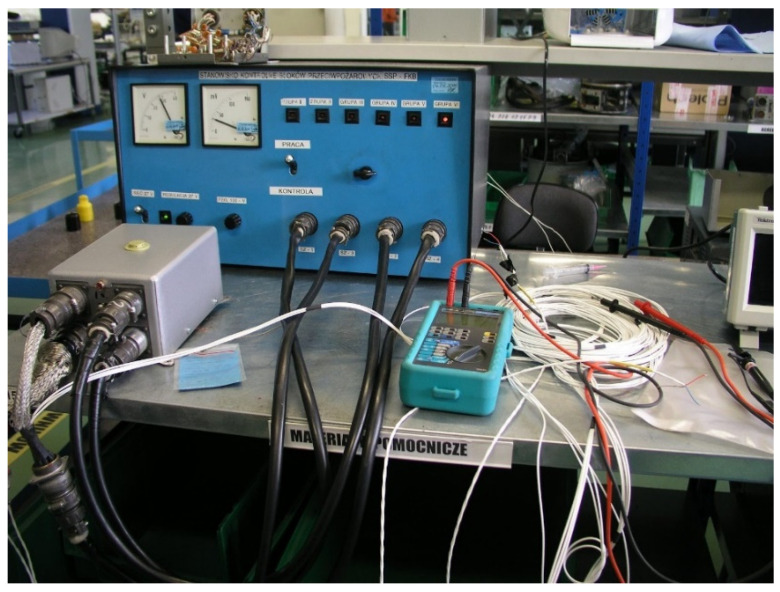
Test bench WZL-1 for checking the correct operation of the SSP−FK−BI executive block s.2. on the “CONTROL” (in Polish “KONTROLA”) range.

The experiment can be summarized in the following steps:

1. Preparation of the measuring system for the WZL−1 test bench (according to Figure 7). Set the operating range of the block in the “OPERATION” position on the test stand;
2. Switch on the power supply of the SSP−FK−BI s.2. actuating block with a direct current of 28.5 V (none of the lamps of the stand should come on);
3. Checking activation of the executive block when a voltage simulating the signal from the DPS sensor is applied. Activation of the appropriate channel of the executive block should occur for a voltage within the range of 28 ÷ 32 mV;
4. Verify the correct functioning of the block;
5. If the block is found to trip at a DPS transmitter voltage of less than 20 mV, check in 2 mV steps, determining the minimum DPS transmitter voltage at which the block trips;
6. For each amplifier board of the SSP−FK−BI s.2., determine thresholds of block tripping and deactivating and deactivation thresholds of the block, causing the RES-5 relay contacts to switch on and off;
7. For each amplifier board of the SSP−FK−BI s.2. block, determine the tripping thresholds and deactivation thresholds of the block that cause activation and deactivation of the RES-52 relay contacts at rising supply voltage;
8. After the test, draw up a diagram of the tripping and release thresholds of the block and compare it with the results obtained during the tests in the laboratory;
9. Proceed with a recheck of the correct operation of the block. If the block is found to operate due to a reduction in the supply voltage at a sensitivity threshold below 20 mV, determine the cause of the increased sensitivity of the block and replace the amplifier boards so operating, considering them to be faulty;
10. If the block is found to operate due to a reduction in the supply voltage at the sensitivity threshold between 20 mV and 28 mV and the tripping band is significantly different from that shown in Figure 7. (the change in the block supply voltage causing tripping is more than 5 V), the reason for the increased sensitivity of the block should be determined, and the amplifier boards operating in this way should be replaced as they are faulty.
11. Damaged boards should be replaced with functioning ones, and after replacement, the correctness of block operation should be checked again.

### 5.2. Simulation Tools

Simulink is a MATLAB-integrated interactive package for the modeling and simulation of dynamic, continuous, and discrete systems without the need to create simulation program code. It allows graphical modeling in the form of block diagrams and physical models using ready-to-use elements downloaded from the package’s libraries.

Applications of Simulink are very broad and include digital signal processing, the analysis of electrical circuits and the design and testing of devices and control systems. The analysis of systems using simulation methods can significantly reduce the time and costs needed to prepare or improve control system prototypes [20].

Circuit-Maker [30] is an advanced software solution for designing electronic circuits and creating simulations of designed circuits, equipped with a powerful component finder, along with access to design elements. The tool allows the study of the behavior of electronic circuits that cannot be tested in real-world conditions and facilitates the planning of experiments in both laboratory and operational settings. Circum-Maker allows also for dynamic modeling, making it possible to test the capabilities of the DPS and DTBG sensors under changeable thermal losses (sudden cell cooling).

## 6. Results

### 6.1. Determination of Static and Dynamic Characteristics of Fire Detectors

To determine the static and dynamic properties of the DPS and DTBG thermoelectric fire sensors, a simulation model of their operation was developed. The simulation model of the fire sensors developed in the MATLAB-Simulink package allows the determination of the instantaneous temperature of the covered and uncovered joints of the first crown (for DPS sensors) and the uncovered joints of the second crown (for DTBG sensors). The model allows the input of changes in ambient temperature (characterizing the thermal conditions of the selected time of day or year), heating temperature (temperature build-up when the KO-50 heating furnace or EWU exhaust gas scatters are switched on) and cooling temperature (temperature drop as the aircraft gains height).

In a constant thermal state, when the temperature of the exposed joints of the fire sensor is the same as the temperature of the covered joints (located in the sensor interface), the generated thermoelectric force from the sensor takes on a value of zero, i.e., the static characteristic of the fire sensor as a function of time is described by a straight line with a value of zero. The dynamic characteristic can be described as a step-like forcing.

**Figure 8:**
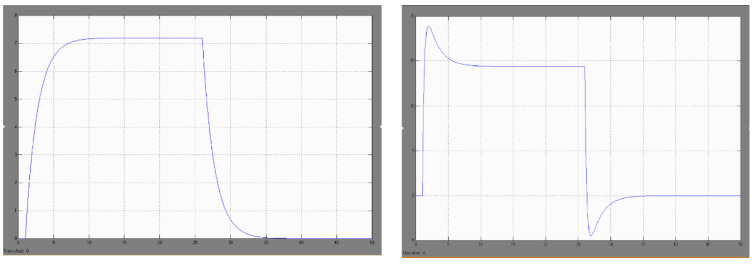
The modeled signal of the thermoelectric sensor after its sudden heating, and subsequent cooling for the DPS sensor (**left**) and for the DTBG sensor (**right**).

In the course of simulation studies in the MATLAB-Simulink package, it was found that in the case of a temperature forcing in the form of a unit stroke (modeling the study of a sudden blast of warm air after switching on the fan of the KO-50 heating furnace) with the assumed process of temperature changes in the air surrounding the uncovered welds of the DPS sensor (Figure 8, view left): −20 C, +60 C, −20 C, the thermoelectric force generated in the sensor is, respectively: 0 mV, +7.2 mV, 0 mV [29].

Investigations carried out have shown that the sudden heating of one DPS sensor does not cause a significant increase in the thermoelectric force generated in the sensor and it cannot be the cause of spontaneous tripping of the SSP−FK system in the assumed thermal conditions (i.e., switching on the KO-50 heating furnace).

Similarly, in the simulation studies of the DPS sensor, it was found that in the case of temperature forcing in the form of a unit stroke with the assumed process of temperature changes in the air surrounding the exposed welds of the DTBG sensor (Figure 8, view right): −20 °C, +60 °C, −20 °C, the thermoelectric force generated in the sensor is, respectively: 0 mV, +18.2 mV/+14 mV, −4.5 mV/0 mV

These tests showed that the sudden heating of one DTBG sensor does not cause a sufficient increase in the thermoelectric force generated in it (up to the threshold value) and it cannot be the cause of spontaneous tripping of the SSP−FK system for the assumed thermal conditions.

In the course of simulation studies of the model of three, grouped, fire sensors, it was found that for the assumed jump in the temperature of the air surrounding the external welds of the sensor in the case of modeling the activation of the KO-50 furnace, with the assumed course of changes in the order: −20 °C, +60 °C, −20 °C, the thermoelectric force generated by the group of three sensors is, respectively: 0 mV, +21.6 mV, 0 mV (for the three DPS sensors) and 0 mV, +54.6 mV/+42 mV, −13.5 mV/0 mV (for the three DTBG sensors). Since the minimum value of thermoelectric force required to trigger the fire alarm system is 26 mV, the three DTBG sensors may be the cause of false tripping of the SSP-FK system due to the sudden application of warm air when the KO−50 heating cooker is switched on (the three DPS sensors generate a subthreshold signal of 21.6 mV, which may be relevant under conditions of supply voltage disturbances).

### 6.2. Modeling the Impact of Sudden Heating and Cooling of Fire Detectors

The modeling of the effect of sudden heating followed by sudden cooling and reheating of the joints of the exposed DPS and DTBG fire detectors is intended to simulate the case of a sudden blast of cold air during the operation of the KO−50 heating furnace (or temperature changes during weld shrouding by the exhaust gases from the EWU scatterers).

**Figure 9:**
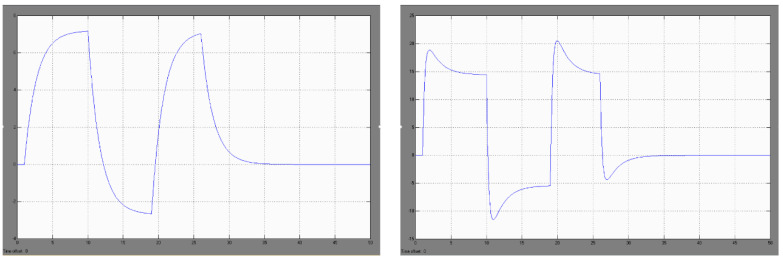
Modeled waveforms of the thermoelectric sensor signal after its sudden heating and cooling during heating for the DPS sensor (**left**) and for the DTBG sensor (**right**).

Simulation studies of the above case have shown that a sudden cooling of the air surrounding the DPS sensor subjected to heating from the KO-50 furnace with the assumed course of temperature changes: −20 °C, +60 °C/−50 °C/+60 °C, −20 °C induces a generation of the thermoelectric force, respectively (Figure 9, view left): 0 mV, 7.2 mV/−2.6 mV/+7.2 mV, 0 mV.

The research has shown that the sudden heating, cooling and then reheating of a single DPS sensor does not cause a significant increase in the thermoelectric force generated in the sensor and cannot cause the SSP−FK system to trip itself for the assumed thermal conditions (i.e., switching on the KO−50 heating furnace).

Analogous investigations, carried out for one DTBG sensor, showed (Figure 9, view right) that the rapid cooling of the air surrounding exposed welds subjected to heating from a KO-50 furnace with an assumed temperature variation: −20 °C, +60 °C/−50 °C/+60 °C, −20 °C induces the generation of thermoelectric force, respectively: 0 mV, 18.2 mV/+14 mV, −12 mV/−5.1 mV, +21 mV/+14 mV, −4.5 mV/0 mV.

The results of these tests showed that the sudden heating, cooling, and reheating of one DTBG sensor does not cause a significant increase in the generated thermoelectric force and cannot be the cause of false tripping of the SSP-FK system for the assumed thermal conditions. At the same time, it should be stated that the generated thermoelectric force, obtained in simulation studies for the assumed thermal conditions, can reach sub-threshold values of 21 mV for the SSP-FK-BI actuator block, for which the minimum tripping value under normal 28.5 V supply conditions is 26 mV.

The obtained results for the DPS and DTBG sensors are the input parameters for the studies of the false trigger of the executive block from disturbing impulses occurring in the power circuits.

### 6.3. Study of the Model with Regard to the Possibility of False Trigger of the Executive Block from Disturbing Impulses Occurring in the Power Circuits

The aim of the simulation studies was to determine which types of supply voltage disturbances and at which sub-threshold signals from the fire sensor cause false tripping of the system. This makes it possible to determine what type of voltage stabilizers or fire sensors should be used to protect the system from the effects of these disturbances.

The basic case in modeling the operation of the SSP−FK system, in which false activation of the system is assumed, is the appearance of momentary drops in the supply voltage during the occurrence of a sub-threshold signal from the fire sensor. The operation of the input path of the amplifier board of the SSP−FK−BI block in such a condition consists of the following. The signal at the inverting input of the US1 amplifier operating in the comparator circuit (which is the reference voltage) is exceeded by the input voltage from the sensor circuit, fed to the non-inverting input of the comparator.

A condition for the operation of the fire alarm system is that the signal from the fire sensor has an instantaneous value greater than the reference signal, reduced when a drop in the supply voltage occurs. This condition is fulfilled when the signal from the fire sensor will have a sub-threshold value, i.e., it will be greater than the minimum starting value, but will not exceed the value that triggers the system for the rated power supply.

To demonstrate the ability to model the operation of the amplifier board under fault conditions, periodic waveforms were used to characterize voltage drops in the board’s power supply with a pulse value of −14.80 V and a duration of 200 ms. The above values became the basic data adopted in the Circuit-Maker program to determine the tripping conditions of the SSP-FK fire protection system at voltage drops and the occurrence of the thermoelectric force generated from the fire sensors. In the simulation scheme built for the operation of the amplifier board, an off-the-shelf component in the form of a voltage source model with an adjustable DC voltage value of 24 mV was used to model the signal from the DPS sensors. A ready-made programmed element in the form of a voltage source model with a triangular waveform and adjustable signal amplitude was used to model changes in the supply voltage of the executive block with amplifier boards.

#### Modeling the Voltage Waveform at the Signal Comparator Output

The purpose of the study in modeling the voltage pattern at the output of the signal comparator US1 was to determine the value of the signal from the fire detector and the parameters of the instantaneous voltage drops of the on-board power supply necessary for the output signal from the signal amplifier US2 to pass to a low level, causing the opening of the power transistor T3 and the self-activation of the amplifier board of the executive block SSP−FK−BI.

In the case where, in the simulation model tested, the fire detector did not generate a thermoelectric force (no fire), a momentary drop in the power supply voltage did not result in a false tripping of the system, regardless of the shape and amplitude of the disturbance pulse occurring in the on-board power supply.

**Figure 10:**
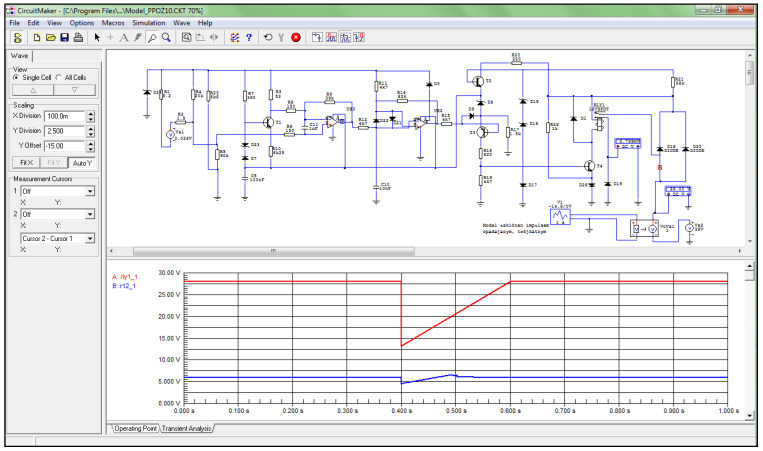
Voltage waveform at the output of signal comparator US1 (blue) for 24 mV.

In the case of a simulation of a sudden increase in thermoelectric power to a subthreshold value of 24 mV (e.g., when the fire sensor in the engine compartment is momentarily heated due to being wrapped in warm air from the EWU gas scatterers), there is an increase in the voltage applied to the non-inverting input of comparator US1 of the amplifier board of this channel (Figure 10), resulting in a false tripping of the system.

Simulation studies have shown that when the voltage from the fire sensor circuit, fed to the non-inverting signal input of the US1 comparator, exceeds the reference voltage, the output voltage from the US1 comparator rises to a high level. Analysis of the voltage waveforms obtained showed that, under normal 28.5 V supply conditions, the output voltage from the US1 comparator changes its value from a low level of 1.34 V to a high level of 7.18 V when a thermoelectric force of 30 mV is generated from the fire sensor.

The test results have confirmed the hypothesis that when the voltage is lowered from the fire sensor, triggering the US1 comparator requires a reduction in the on-board power supply voltage. For an assumed thermoelectric force value of 24 mV, occurring during a sudden change in the on-board supply voltage caused by switching on a high-power receiver, the output voltage from the comparator changes from a low level of 1.34 V to a high level of 6.44 V, but tripping of the actuator block only occurs for supply voltages between 14.60 V and 20.80 V.

In an analogous procedure, signal waveforms were determined for the signal amplifier US2, transistor T4, which made it possible to model the waveform of the voltage at the output of the RES−52 executive relay built on the amplifier board. Based on the simulated waveform, the parameters of the signal from the smoke detector were determined, as well as the momentary voltage drops of the on−board power supply necessary to close the minus circuit of the RES−52 output relay coil and the automatic operation of the SSP−FK−BI executive block amplifier board.

**Figure 11:**
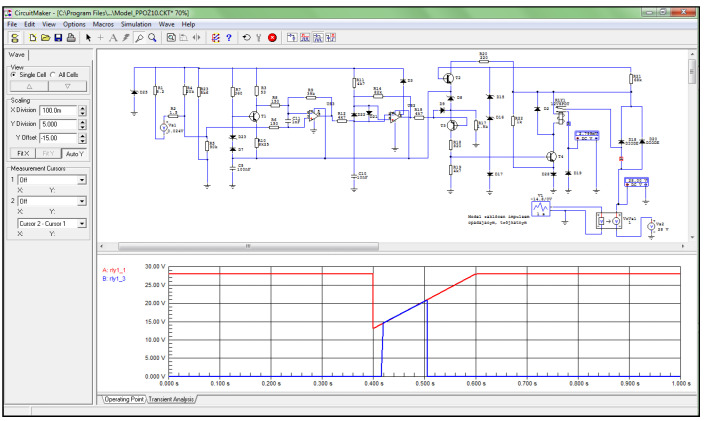
Voltage waveform at the RES−52 relay output (blue) for 24 mV.

In the case of simulating a sudden increase in thermoelectric power from the sensor to a subthreshold value of 24 mV, there is an increase in the voltage applied to the base of transistor T4 and further closure of the minus circuit of the RES−52 output relay coil, which causes a current to flow in it and close the contacts for the fire signaling circuit (Figure 11), resulting in a false activation of the system.

Simulation tests have shown that the activation of transistor T4 causes the minus circuit of the RES−52 output relay to be closed by diode D28, which, when energized, causes the circuit to be closed and the fire signal to be generated. Activation of the RES−52 relay causes supply voltage to be applied to terminal 2 of the amplifier board of the selected channel of the SSP−FK−BI executive block, from which the fire signal is received in the supervised compartment. This signal is fed further to the executive circuits that activate the fire signaling and extinguishing system.

Analysis of the voltage waveforms obtained showed that under normal on−board supply conditions of 28.5 V, when the thermoelectric force is 30 mV, the voltage value on the collector of transistor T4 changes from a high level of 27.80 V to a low level of 1.20 V, which causes current to flow through the coil of the RES−52 relay and close its contacts. When the supply voltage is reduced, during thermoelectric force generation from the 24 mV sensor, the voltage in the minus circuit of the RES−52 relay coil when it is activated reaches values between 1.20 V and 1.18 V, but block tripping only occurs for supply voltages that are between 14.60 V and 20.80 V.

**Figure 12:**
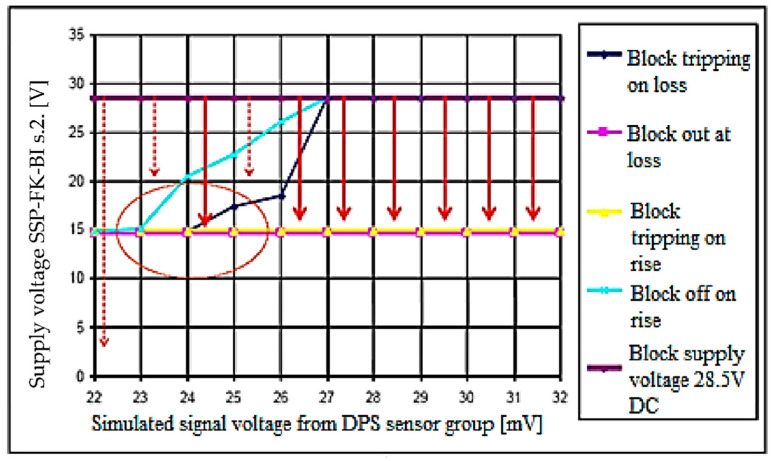
Theoretical response and holding characteristics of SSP−FK−BI block.

Based on the simulation results obtained regarding the value of the fire sensor signal required for tripping the SSP−FK−BI executive block when the voltage drops in its power supply circuit, a summary diagram (Figure 12) was developed, showing the block’s tripping area and the area of sustained tripping, with a supply voltage of 28.5 V.

## 7. Experimental Studies of SSP−FK−BI Executive Blocks under Electrical Disturbance Conditions

In the course of testing the level of interference occurring in the power circuits of the SSP−FK system performed on the Mi−8 helicopter, it was found that at the moment of switching on and off power sources (e.g., the GS−18TP starter generator) and high−power receivers (e.g., the KO−50 heating furnace), interference impulses appear. The parameters of these pulses can reach values of +2.8 V/0.1 s and −14.8 V/1 s (when switching on the GS−18TP) and +21 V/1 s (when switching off the GS−18TP). In the system control circuits, interference was found with levels approximately 10 times lower, i.e., +0.2 V/0.01 s and −1.4 V/0.1 s (when switching GS−18TP on) and +2.1 V/0.1 s (when switching GS−18TP off).

**Figure 13:**
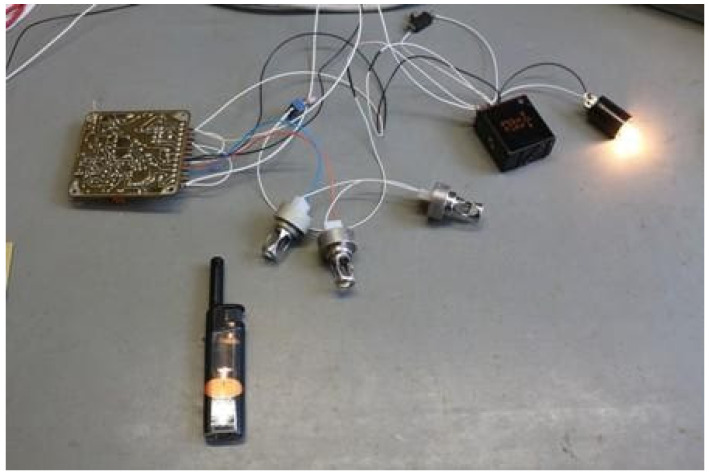
View of the measuring system for testing the block for disturbances in the supply voltage.

Based on the obtained test results, a model of the out−of−current interference pulse generator (Figure 13) was developed and built, and tests were performed on the SSP−FK fire protection system under laboratory conditions. It was found that momentary drops in the supply voltage of the amplifier board of the order of 14.8 V, with the presence of a subthreshold voltage from the fire sensor of the order of 24 mV, were the cause of its false tripping, and that the characteristics of the amplifiers included zones of sustained tripping [9].

Overvoltage pulses represent another group of disturbances that were introduced into the circuit as momentary changes in the supply voltage of the amplifier boards. Laboratory tests showed that pulses with an amplitude of up to 50 V and a duration of up to 10 µs are too short to trigger the RES−52 internal relay, even though the amplitude value at the base of transistor T4 is sufficient to open it (it closes the RES−52 relay supply circuit). However, for a longer−lasting disturbance pulse (with a period of the order of 10 ms), the required voltage value at the base of transistor T4, capable of triggering the RES−52 relay and generating a fire signal, was not found.

Experimental tests confirmed that one of the possible causes of the false tripping of the SSP−FK fire protection system is the occurrence of local short circuits between the circuits on the amplifier board. The “board power supply—base of transistor T4” circuit contains electronic components (e.g., capacitors and transistors) that constitute filter circuits. As a result of their short−circuit due to moisture, the filtering properties are temporarily lost and the electrical impulses occurring in the power supply circuits of the amplifier boards can trigger the fire alarm system. Laboratory tests showed that for an interference impulse amplitude of 30 V and a duration of 10 ms, there is an opening voltage at the base of transistor T4, which activates the RES−52 relay. This confirms the hypothesis on the possibility of false tripping of the SSP−FK system because of short circuits and momentary overvoltage occurring in the on−board power supply network.

The results of the investigations proved that, under normal on−board power supply conditions of 28.5 V, the SSP−FK−BI executive block generates an output signal, activating the fire suppression system, for an input voltage from the fire sensor with a threshold value of 30 mV.

At the same time, they confirmed the possibility of false triggering of the executive block in the event of momentary drops in the on−board power supply voltage, with the thermoelectric force from the fire sensors having a sub−threshold value (less than 30 mV).

**Figure 14:**
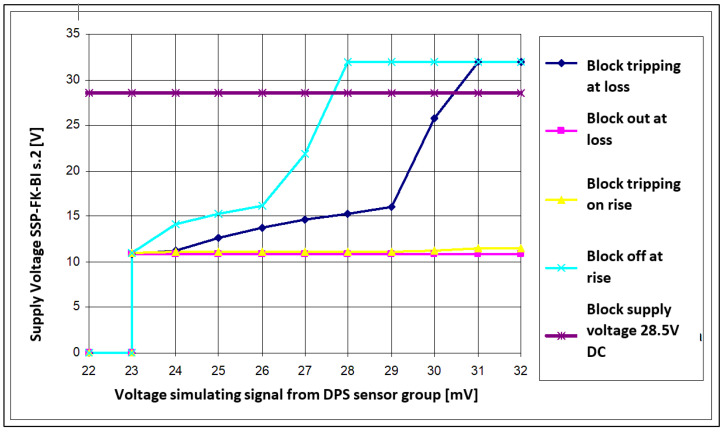
Actual trip and hold−up characteristics of SSP−FK−BI block.

On the basis of the measurement results obtained with regard to the value of the signal from the fire sensor required for activation of the SSP−FK−BI executive block with a voltage drop in its supply circuit, a summary diagram (Figure 14) was developed, showing the block’s tripping area and the area of sustained tripping. The graph determined in the study (Figure 14) allows confirmation of the possibility and determination of the conditions of self−acting activation of the SSP−FK fire protection system when performing a ‘cold’ or ‘hot’ start−up of the Mi−8 helicopter’s engine, as well as at the moment of switching on the high−power receivers.

This graph shows that actuation of the actuator block can occur for fire sensor signals smaller than the manufacturer’s stated values for the rated supply voltage (28 ÷ 32 mV). It also helps to understand why not every drop of voltage in the electrical supply causes the block to trip.

To cause false tripping of the block, the voltage drop must have values within the tripping range and, once in the tripping hold area, it must last long enough for the block’s self−sustaining power systems to switch over. The trip hold area allows the block to start for supply voltage drop times shorter than those required in the trip area.

The results obtained showed that the existence of the tripping hold area of the executive block occurs as an extension of the tripping actuation area and starts for fire sensor signals of approximately 23 mV.

## 8. Conclusions

The fire suppression system, built into the aircraft, plays a very important role in ensuring flight safety. A false activation of this system results in an extremely dangerous and stressful situation for the crew every time, interrupting the task at hand and landing, usually in rough terrain. The discharge of the extinguishing agent, because of false activation of the system, can be the cause of a major failure or catastrophe in the event of a real fire on board the aircraft.

Simulation studies of numerical diagnostic models of the SSP−FK fire protection system were carried out at the Air Force Institute of Technology, which make it possible to determine the conditions causing its false tripping and to determine parameters for selected disturbing factors. Computer−aided testing can also be used to design specialized control and measurement systems designed to generate electrical disturbances that may be the cause of false tripping of the SSP−FK fire protection system. Numerical and experimental studies were based on analyses of the operation of the SSP−FK fire protection system. They covered the system operated on board the Mi−8 family of helicopters (utilizing the SSP−FK−BI executive block) and An−28 aircraft (using the BI−2A executive block). The results of the analysis made it possible to identify the characteristics in terms of architecture, operating ranges, and mode of operation of the SSP−FK system, and thus the diagnostic symptoms of the selected for simulation and experimental studies

The simulation test methods used at the Air Force Institute of Technology complement the diagnosis process of airborne fire protection systems and allow the number of false triggers to be reduced to a minimum. For operational needs, including the technical support of the Air Force Engineering Service, they form the basis for the development of a so-called comprehensive test bed for the SSP-FK fire protection system.

The effect of the simulation studies is to determine what type of isolation resistance, and between which points of the printed circuit board of the amplifiers, cause false tripping of the system.

## Figures and Tables

**Table 1:** Number of failure cases.

Number of An-28 aircraft in service	8
Number of aircraft in which fire protection system damage occurred.	3
Number of fire system component failures.	5

## Data Availability

Not applicable.

## References

[1] Tooley M.H., Wyatt D. (2011). Aircraft Electrical and Electronic Systems: Principles, Operation and Maintenance.

[2] Zafar R., Zaib S., Asif M. (2020). False Fire Alarm Detection Using Data Mining Techniques. Int. J. Decis. Support Syst. Technol..

[3] Avazov K., Mukhiddinov M., Makhmudov F., Cho Y.I. (2022). Fire Detection Method in Smart City Environments Using a Deep-Learning-Based Approach. Electronics.

[4] Guo S., Eriksen H., Childress K., Fink A., Hoffman M. High Temperature High Accuracy Piezoresistive Pressure Sensor Based on Smart-Cut Soi. Proceedings of the 2008 IEEE 21st International Conference on Micro Electro Mechanical Systems.

[5] Scheffey J., Darwin R., Leonard J. (1995). Evaluating Firefighting Foams for Aviation Fire Protection. Fire Technol..

[6] Snyder B.L., Anderson K.J., Renken C.H., Socha D.M., Miller M.S., Pirich A.R., Hayduk M.J., Donkor E. (2004). Multi-Sensor Cargo Bay Fire Detection System. Proceedings of the Enabling Photonic Technologies for Aerospace Applications VI.

[7] Pathak A., Norrefeldt V., Pschirer M. (2021). Validation of a Simulation Tool for an Environmentally Friendly Aircraft Cargo Fire Protection System. Aerospace.

[8] Szelmanowski A., Zieja M., Pazur A., Głyda K. (2020). Studying the Dynamic Properties of an Amplifier Board Execution Block in Terms of False Tripping of an Aircraft Fire Suppression System. Engineer of the XXI Century.

[9] Wang Y., Yu C., Tu R., Zhang Y. (2011). Fire Detection Model in Tibet Based on Grey-Fuzzy Neural Network Algorithm. Expert Syst. Appl..

[10] Wang Y., Zhang Q., Soutis C., Gresil M. (2021). Development of a Fire Detection and Suppression System for a Smart Air Cargo Container. Aeronaut. J..

[11] Yue N., Li Y., Bai M., Hou S., Gizardin M., Zhang S., Ding S. (2014). Flammable Fluid Fire Protection Airworthiness Design and Verification Method of Civil Transport Aircraft. Proceedings of the 3rd International Symposium on Aircraft Airworthiness (isaa 2013).

[12] Liu R., Yuan C., Ma W., Liu S., Lu S., Zhang H., Gong J. (2022). Simulation Study on Aircraft Fire Extinguishing Pipeline with Different Filling Conditions and Pipeline Characteristics. Fire.

[13] Hamdan M. (2014). Automatic Fire Fighting System. IOSR J. Eng..

[14] Sholanke A., Oche A., Paul O., Taylor G. (2020). Fire Emergency Safety Preparedness in the College of Leadership Development Studies Building in Covenant. Civ. Eng. Archit..

[15] Szelmanowski A., Zieja M., Głyda K. (2017). Dynamic Properties Modeling of the Thermoelectric Fire Sensors in the Aircraft Fire Suppression System. J. KONBiN.

[16] (2007). Fire Protection Systems for Boeing P-8A. Aircr. Eng. Aerosp. Technol..

[17] Festag S. (2016). False Alarm Ratio of Fire Detection and Fire Alarm Systems in Germany—A Meta Analysis. Fire Saf. J..

[18] U.S. Department of Transportation, Federal Aviation Administration (2008). Aircraft Cargo Compartment Multisensor Smoke Detection Algorithm Development.

[19] Driessche M.V.D. (2019). Air China Flight from Beijing to Los Angeles Makes an Emergency Landing in Siberia. https://www.aviation24.be.

[20] Danish M., Luo S. (2020). A New Route to Enhance the Packing Density of Buckypaper for Superior Piezoresistive Sensor Characteristics. Sensors.

[21] Leal-Junior A., Frizera-Neto A., Marques C., Pontes M.J. (2018). A Polymer Optical Fiber Temperature Sensor Based on Material Features. Sensors.

[22] Skowroński G. (2003). An-28 w Polsce. Lot. Z Szachownica Ilustrowany Mag. Milos. Hist. Lotnictwa Pol..

[23] Polskie Zakłady Lotnicze (2001). An-28 “Bryza” Aircraft Flight Manual.

[24] Frawley G., Thorn J. (1995). The International Directory of Civil Aircraft.

[25] Cimerman F., Blagojevic B., Bajsic I. (2002). Identification of the Dynamic Properties of Temperature-Sensors in Natural and Petroleum Gas. Sens. Actuator A Phys..

[26] Moir I., Seabridge A. (2004). Design and Development of Aircraft Systems: An Introduction.

[27] Głyda K., Szelmanowski A. (2017). Study of properties of the SSP-FK aircraft fire suppression system in terms of false activation. ZN PRz Mech..

[28] Szelmanowski A., Głyda K., Gajewski T., Tokarski T., Kalisiak A., Sekuła A. (2015). Testing the Possibility of Automatic Activation of the SSP-FK Fire Protection System of Helicopter Mi-8 No. 660 in the Event of a Power Failure/Voltage Drop.

[29] Głyda K. (2022). Testing the Characteristics of the Airborne Fire Protection System. Ph.D. Thesis.

[30] Fabio A. CircuitMaker from Altium. https://hackaday.com/2014/09/24/circuitmaker-from-altium/.

